# Anesthetic Challenges of Caring for an Adult With Moebius Syndrome: A Case Report

**DOI:** 10.1155/cria/1908098

**Published:** 2025-11-21

**Authors:** Geetasravya Vegunta, Giovanna Patafio, Swata Gade, Kwame Awuku, Evelyne Kalyoussef, Shridevi Pandya Shah

**Affiliations:** ^1^ Department of Anesthesiology, Rutgers New Jersey Medical School, Newark, New Jersey, USA, rutgers.edu; ^2^ Department of Otolaryngology, Rutgers New Jersey Medical School, Newark, New Jersey, USA, rutgers.edu

**Keywords:** case report, difficult airway, Moebius syndrome

## Abstract

**Background:**

Moebius syndrome (MBS) is a rare congenital disorder with facial nerve palsies and craniofacial malformations, increasing the risk of a difficult airway during anesthesia. We report a difficult airway in an adult with MBS during an elective procedure, which is unique as most reports of MBS are in children.

**Case Presentation:**

Our patient was a 30‐year‐old male diagnosed with MBS at birth and presented with craniofacial and orthopedic malformations, intellectual disability, and facial nerve palsies. Although our patient had prior successful intubations, their airway exam deteriorated from Mallampati Classes II to IV after many surgeries. After fiberoptic intubation with nebulized lidocaine to maintain spontaneous ventilation failed, an attempt with dexmedetomidine and video laryngoscopy was successful.

**Conclusion:**

Our case demonstrates the necessity of preparing for a difficult airway and strategies for airway management in an adult with MBS.

## 1. Introduction

Moebius syndrome (MBS) is a congenital developmental disorder characterized primarily by facial nerve paralysis or palsy and frequently associated with cranial neuropathies, craniofacial malformations, cardiac defects, and orthopedic anomalies [1–10] (Table 1). The etiology of MBS is unknown, but theories include embryological defects with spatial and temporal brain development, interruption of vascular supply to the brain, or teratogen exposure during pregnancy [1–4]. Appropriate anesthetic management is imperative as patients with MBS undergo multiple corrective or palliative surgeries [2, 3, 5]. In this report, we present a difficult intubation in an adult with MBS.

**Table 1:** Complications associated with Moebius syndrome [1–10].

Complications associated with Moebius syndrome	
Craniofacial	Micrognathia, cleft palate, mandibular hypoplasia, retrognathia, trismus, epicanthal folds, hemifacial microsomia, entropion
Cranial neuropathies	II, III, IV, V, VI, VII, VIII, IX, X, XI, XII
Cardiovascular	Ventricular septal defect, atrial septal defect, patent ductus arteriosus, dextrocardia, transposition of the great vessels, total anomalous pulmonary venous return
Orthopedic	Scoliosis, talipes, syndactyly, congenital limb and digit amputations, pes planus, valgus femur, Poland syndrome, Klippel–Feil syndrome, facioscapulohumeral muscular dystrophy
Neurological	Intellectual delay, developmental delay, seizures, hypotonia, hyperreflexia, cervical hydromyelia, autism
Miscellaneous	Gastroschisis, hypospadias, hydronephrosis, obesity, laryngeal palsy, pharyngeal palsy

## 2. Case Description

A 30‐year‐old male, diagnosed with MBS at birth, presented for an elective septoplasty, endoscopic turbinate resection, and Jones tube placement. A preoperative physical exam revealed a 44.9‐kg, 144.8‐cm male with intellectual disability, vision and hearing deficits, craniofacial deformities, right facial paresis, right upper and lower extremity weaknesses, kyphoscoliosis, and a short thick webbed neck. Cardiac, pulmonary, and abdominal exams were within normal limits. An airway exam revealed a limited and left‐deviated mouth opening with trismus and a class IV Mallampati airway. Previous surgical history documents patient’s airway as Mallampati Class II without airway. Due to concern for difficult airway, the possibility of a tracheostomy was discussed with the patient and family.

Initially, the anesthetic plan was to perform fiberoptic intubation while maintaining spontaneous ventilation. A size 6.0 endotracheal tube was chosen based on the patient’s size. A McGrath was readily available for video laryngoscopy and the surgical team was present at induction and prepared to perform a surgical airway if necessary. Preoperatively, a 20‐gauge peripheral IV was placed. The patient was brought into the operating room. The patient’s body habitus, inability to follow commands, and right‐sided weakness made it difficult to obtain an optimal supine position on the operating room table. Standard American Society of Anesthesiologist monitors were placed, and vitals were noted to be within normal limits. A nasal cannula was placed for oxygen administration before initiating sedation. The patient was administered midazolam 2 mg intravenously (IV) as an anxiolytic and 0.2 mg of glycopyrrolate IV as an antisialagogue, and the airway was anesthetized with nebulized 4% lidocaine. Dexmedetomidine was initiated with a bolus of 23 mcg (0.5 mcg/kg) over 3 min, followed by a 0.5 mcg/kg/hr infusion for sedation. Once adequate sedation was achieved, fiberoptic intubation was attempted. The patient’s copious secretions, strong cough reflex, and anterior and deviated airway made the fiberoptic approach difficult, so the patient quickly desaturated to SpO_2_ of 80. Therefore, fiberoptic intubation was aborted, and supplemental oxygen was applied with a face mask. The patient’s oxygenation status improved and the infusion rate of dexmedetomidine was increased to deepen sedation.

Since prior intubations were successful with video laryngoscopy using either a cMAC or McGrath, video laryngoscopy with a McGrath Size 3 blade was attempted. With cricoid pressure a Grade IIb Cormack–Lehane view was obtained. The patient’s head was extended and the blade was placed midline with a partial view of the epiglottis and cords to give more space for tube passage. Placement was confirmed with capnometry and auscultation. A 100 mg of propofol IV to deepen the anesthetic and 30‐mg rocuronium IV for paralysis was administered. Then the patient was placed on synchronized intermittent mandatory ventilation (SIMV) with pressure support. Anesthesia was maintained throughout the case with inhaled anesthetic, sevoflurane, and 0.5 mcg/kg/hr dexmedetomidine infusion. A lower body forced‐air patient rewarming system was placed, and normothermia was maintained. A total of 750 mg of acetaminophen IV was given for pain control. A total of 4 mg of ondansetron IV and 8 mg of dexamethasone IV were administered to prevent postoperative nausea and vomiting. Before extubating, the patient had 4/4 twitches, and neuromuscular blockade was reversed with 200 mg of sugammadex IV. Spontaneous ventilation with adequate tidal volumes was noted. The patient was successfully extubated, and the postoperative period was uneventful.

## 3. Discussion and Conclusions

There are many documented cases discussing MBS and related anesthetic considerations in the pediatric population [3, 5–8]. However, there is limited literature about anesthetic care for adults with MBS. Our case is unique in that we describe MBS in an adult with a progressively difficult airway. In one documented case, a 33‐year‐old female with MBS with adequate mouth opening and without craniofacial deformities was managed with an laryngeal mask airway without difficulty [11]. In another case, fiberoptic intubation was planned on a 23‐year‐old male with MBS and a sigmoid volvulus; however, the patient unexpectedly became hemodynamically unstable with induction drugs, developed cardiac arrest, and failed both fiberoptic and direct laryngoscope intubation [12].

Difficult airways are infrequent in patients with MBS [2]. However, several components of MBS can contribute to airway difficulties, making a preoperative exam essential. Craniofacial abnormalities associated with MBS including retrognathia, micrognathia, and cleft palate may result in anatomical variations, such as restricted mouth opening, causing challenges in airway management[2, 4–6]. Despite prior uncomplicated intubations, our patient’s history, airway exam, body habitus, trismus, facial paralysis, and craniofacial dysmorphia raised concerns for a difficult airway. The airway exam worsened from a previously documented Mallampati Class II to a Mallampati Class IV, likely attributed to the numerous surgeries our patient underwent [6]. While MBS is not progressive, repeated surgeries can lead to scarring and fibrosis reducing tissue mobility and mouth opening. In addition to standard preoperative history and physical exam, it is important to review previous anesthesia notes to guide plans, specifically noting history of tracheostomy, aspirations, feeding difficulties, and other associated syndromes. Airway should be evaluated for mouth opening, neck mobility and extension, nasal patency, dental status, and Mallampati score. The patient’s ability to close and protect eyes as well as swallow and gag reflex should be assessed as well. The patient’s baseline breathing and cardiac exam should also be noted.

To ensure a safe and secure airway could be obtained multiple contingencies were prepared. Video laryngoscopy, fiberoptic bronchoscopy, and tracheostomy equipment was readily available in the operating room.

Though the anesthetic plan included fiberoptic intubation in anticipation of a difficult airway, several aspects of the patient’s presentation made implementing this modality challenging. His craniofacial abnormalities resulted in anterior and deviated airway structures. In MBS, palsies of cranial nerves V, VI, VII, IX, X, and XII affect facial muscle and lingual or pharyngeal motor function and coordination. This increases the likelihood of regurgitation, inadequate secretion control, or aspiration [3, 5–7]. Patients should be closely monitored for secretions, and antisialogogues administration should be considered [2]. Despite administering glycopyrrolate, the patient produced copious secretions that impaired visualization of the airway with the fiberoptic scope. The team also encountered difficulty in positioning and directing the patient. MBS is associated with varying degrees of intellectual disability [3–5, 8], and our patient had an intellectual delay consistent with a third‐grade level. The combination of intellectual disability and preoperative anxiety provided challenges in communicating with our patient during induction, resulting in an unsuccessful fiberoptic intubation. Positioning the patient in reverse Trendelenburg to allow the secretions to drain away and using a continuous flexible suction alongside the scope can help clear secretions. In addition, preparations should be made for surgical airway and the possibility should be discussed with the patient and family beforehand if intubation is not possible. Micrognathia and abnormal neck anatomy can make surgical access harder and should be considered if tracheostomy is needed.

Anatomical abnormalities associated with MBS should also be considered in an anesthetic plan. Abnormalities relating to the cervical spine [5, 7] or lower placement of the larynx can affect the method of intubation and necessary equipment. Orthopedic extremity changes can require additional care in obtaining vascular access and patient positioning [6, 9]. Conditions like talipes, congenital limb loss, or syndactyly [1, 4, 6–9] should be anticipated in patients with MBS. These limb abnormalities may restrict IV access [6] or require careful positioning. Our patient had significant kyphoscoliosis, making proper positioning and cushioning a challenge. Intubation attempts were hindered as a sniffing position was not possible with our patient’s spinal anatomy. Patients with MBS should also undergo a cardiac preoperative workup [6] when necessary. Though not present in our patient, congenital heart abnormalities such as ventricular septal defect or patent ductus arteriosus have been associated with MBS [1, 5] and can alter the anesthetic plan.

Significant pharmacological considerations should be addressed in patients with MBS. Autonomic disturbances such as central hypoventilation and idiopathic tachypnea [5, 7] can be exacerbated in MBS. Opioids were not administered in our case, but if necessary, a judicious [5] approach to opioid use to mitigate these risks is essential. Prior to extubation, adequate neuromuscular blockade reversal must be achieved. In our case, we ensured proper neuromuscular blockade reversal using sugammadex, confirmed by observing four strong twitches with a train‐of‐four monitor and adequate tidal volumes during spontaneous ventilation. It is crucial to consider the risk for malignant hyperthermia (MH) in patients with MBS. Hypotonia, a characteristic associated with MBS [5, 7, 9], raises concerns for MH due to its link with congenital myopathies. MH triggers are commonly used inhalational anesthetics and depolarizing muscle relaxants [10]. Despite our patient not displaying hypotonia or signs of myopathy, a detailed history was obtained before formulating the anesthetic plan.

Postoperative care for MBS should be considered as recovery can be more complex. Patients are at increased risk for airway collapse due to swelling after surgery. It is difficult to reintubate if needed due to swelling and reduced mouth opening as well. Therefore, postoperative ICU monitoring or delayed extubation should be considered in high‐risk cases. If prolonged ventilation is anticipated, some patients may benefit from planned tracheostomies. Patients are also at increased aspiration risk, so aggressive suctioning and semiupright positioning is imperative. Speech and swallow evaluation prior to resuming oral intake should be considered. Opioids can worsen respiratory depression, so multimodal pain with NSAIDS and acetaminophen should be used to minimize opioid use, and opioids should be titrated carefully, and patients should be monitored closely. Patient’s may have difficulty communicating pain and needs due to intellectual disability and lack of facial expression, so communication methods should be established prior to surgery (writing on a board, pointing, etc.).

MBS has many significant anesthetic considerations. Associated craniofacial malformations and cranial nerve palsies affect airway access and maintenance. Orthopedic anomalies require specific positioning, and any cardiovascular defects must be evaluated. In our case, the primary anesthetic concern was airway access and management. The team prepared for a difficult airway upon noting the patient’s craniofacial defects, body habitus, and Mallampati Class IV airway. We demonstrate the imperative need to anticipate and prepare for airway difficulties in patients with MBS. Most reported cases of anesthetic management in MBS are in children. However, adults with MBS should be carefully optimized and monitored for relevant anatomic or physiologic changes that may develop between procedures.

**Nomenclature**
MBS: Moebius syndrome
IV: Intravenous
SIMV: Synchronized intermittent mandatory ventilation
MH: Malignant hyperthermia

## Ethics Statement

This article adheres to the applicable Enhancing the Quality and Transparency of Health Research Case Report guidelines. As the patient is intellectually disabled and was not able to read, informed consent and Written Health Insurance Portability and Accountability Act authorization for the writing and publication of this case report was obtained via proxy. The patient’s parents acted as proxy as the patient was unable to provide verbal consent.

## Disclosure

A preprint has previously been published [13].

## Conflicts of Interest

The authors declare no conflicts of interest.

## Author Contributions

Geetasravya Vegunta helped with literature review, writing the manuscript, and reviewing and revising the final manuscript. Giovanna Patafio helped with literature review, writing the manuscript, and reviewing and revising the final manuscript. Swata Gade helped with literature review, writing the manuscript, and reviewing and revising the final manuscript. Kwame Awuku helped with reviewing and revising the initial draft and final manuscript. This author was also the anesthesia resident in the case. Evelyne Kalyoussef helped with reviewing and revising the final manuscript. This author was also the attending otolaryngologist for this case. Shridevi Pandya Shah helped with writing the manuscript and reviewing and revising the final manuscript. This author was also the attending anesthesiologist for this case.

## Funding

No funding was received for this manuscript.

## Data Availability

This is a case report and so there are no statistical data associated with this article.
